# Intensified chemotherapy and simultaneous treatment with heparin in outpatients with pancreatic cancer – the CONKO 004 pilot trial

**DOI:** 10.1186/1471-2407-14-204

**Published:** 2014-03-19

**Authors:** Uwe Pelzer, Andreas Hilbig, Jens M Stieler, Marcus Bahra, Marianne Sinn, Bernhard Gebauer, Bernd Dörken, Hanno Riess

**Affiliations:** 1 Department of Hematology/Oncology, CharitéCentrum für Tumormedizin, Charité – Universitätsmedizin Berlin, Augustenburger Platz 1, Berlin 13353, Germany; 2 Klinik für Hämatologie und Onkologie, St. Marien-Hospital, Hamm, Germany; 3 Visceral- und Transplantationschirurgie, Klinik für Allgemein, Charité – Universitätsmedizin Berlin, Berlin, Germany; 4 Klinik für Radiologie, Charité – Universitätsmedizin Berlin, Berlin, Germany

## Abstract

**Background:**

Advanced pancreatic cancer (APC), beside its high mortality, causes the highest rates of venous thromboembolic events (VTE). Enoxaparin, a low molecular weight heparin (LMWH), is effective in prevention and treatment of VTE. Some small studies indicated that this benefit might extend to patients with cancer and probably prolong survival due to independent mechanisms. We initiated this safety investigation to get feasibility information on intensified chemotherapy combined with LMWH in outpatients with APC treated in 1^st^ line.

**Methods:**

The trial was a prospective, open-label, single center investigation in outpatients with inoperable pancreatic cancer who were treated with intensified first-line chemotherapy along with concomitant application of subcutaneous LMWH. The combined chemotherapy consisted of gemcitabine 1 g/m^2^ (30 min), 5-FU 750 mg/m^2^ (24 h), folinic acid 200 mg/m^2^ (30 min), and Cisplatin 30 mg/m^2^ (90 min) on day 1 and 8; q3w for the first 12 weeks (GFFC) followed by gemcitabine alone in patients without cancer progression. The simultaneous application of prophylactic enoxaparin started on day 1 of chemotherapy with a fixed dose of 40 mg daily. Statistical analyses were performed using R 3.01 with software package CMPRSK and SPSS software v19.0.

**Results:**

The investigation was stopped after recruitment of 19 patients. At this time 15 patients had completed the required 12 weeks of treatment. Based on 71 cycles of GFFC + enoxaparin (median 4/pt [range: 2–4]) and 108 cycles of single-agent gemcitabine + enoxaparin (median 4/pt [range: 0–18]) the cumulative frequency of NCI-CTC toxicities grade 3/4 was below 10%. One case (5%) of a symptomatic non-lethal thromboembolic event was observed while receiving LMWH treatment. No severe bleeding event as defined in the protocol has been observed. The median overall survival was 10.05 [95% CI: 8.67-18.14] months.

**Conclusions:**

The addition of enoxaparin to GFFC chemotherapy is feasible, safe and does not appear to affect the efficacy or the toxicity profile of the chemotherapy regimen in patients with advanced pancreatic adenocarcinoma. Based on these findings we have initiated the randomized CONKO-004 trial to examine whether enoxaparin reduces the incidence of thromboembolic events or increases overall outcome.

**Trial registration:**

Clinical Trials NCT01945879.

## Background

Pancreatic adenocarcinoma is an aggressive cancer type with early extensive local invasion, rapid systemic spread combined with a high resistance to chemotherapy. This is accompanied by a case fatality rate of 90% and thus constitutes the fourth most-frequent cause of death from solid cancer in the western world [1]. In patients with non-resectable advanced pancreatic cancer (APC) median overall survival without effective systemic anticancer treatment is not higher than 4 months [2-4]. Despite intensive anticancer research in the last decade, five year overall survival in patients with APC is still less than 5% [5]. Single agent gemcitabine (GEM) has become the standard first-line chemotherapy for pancreatic cancer 15 years ago [2]. More recent trials using combination regimens with or without gemcitabine showed improvements in cancer control-rate and even survival advantages, most pronounced for patients with better performance status [6-9]. Thus, patients with good performance status are indicated for intensified treatment with combined chemotherapy regimens [9,10].

Venous thromboembolic events (VTE) are considered to be a commonly accurring major lethal complication in cancer patients. Population based case–control trials display a cumulative incidence of VTE of up to six-fold in cancer patients [11]. Cancer types with highest incidence rates are advanced malignancies of the brain, pancreas, lung, and stomach [12-14]. As compared to more limited stages metastatic disease results in a 4 to 13-fold elevated VTE risk, which is further increased by systemic anticancer therapy [14]. The bidirectional interaction between cancer and hemostasis not only leads to activation of blood cells and the coagulation system resulting in clinically relevant thromboembolism but these processes are also under suspicion to enhance cancer growth and metastatic spread [15]. VTE are considered to be a prognostic-negative factor [11,12] and small studies showed astonishing survival advantages using heparin as prophylactic treatment to prevent VTE [16]. Based on these assumptions our CONKO study group planned to conduct a randomized trial to investigate the impact of low molecular weight heparin (LMWH) in a prospective setting in patients with advanced pancreatic cancer undergoing first line therapy, the CONKO-004 trial [17]. During the preliminary stages we had to undertake a pilot trial to get information on safety and feasibility of combined chemotherapy with simultaneous treatment of the LMWH enoxaparin in patients with advanced pancreatic cancer who are at high risk of gastrointestinal bleeding due to local cancer spread. The toxicity profile, feasibility, and maximum tolerable dosage of the combination of gemcitabine/5-FU/Folinic Acid/Cisplatin was investigated in a past phase-I trial of our study group [18].

## Methods

### Design and treatment

The trial was a prospective, open-label, single center investigation in patients with advanced pancreatic cancer who were treated with first-line chemotherapy in an outpatient setting. The intensified treatment consisted of gemcitabine 1 g/m^2^ (30 min), 5-FU 750 mg/m^2^ (24 h), folinic acid 200 mg/m^2^ (30 min), and cisplatin 30 mg/m^2^ (90 min) on day 1 and 8; q3w. The concomitant application of enoxaparin started on day 1 of chemotherapy with a fixed dose of 40 mg daily until cancer progression. Beyond the initial 3 months of intensified 1^st^-line chemotherapy all patients without cancer progression received further treatment with single agent gemcitabine and enoxaparin to prevent patients from cumulative toxicities. Dose adjustment for enoxaparin was recommended in patients with impaired kidney function or thrombocytopenia within the study according to NCI-CTC (National Cancer Institute Common Toxicity Criteria) guidelines to minimize bleeding risk. Dose adjustment for chemotherapy dosage was realised by protocol-defined regulations [18]. Prophylactic antiemetic therapy and supportive care were provided according to individual symptoms and demand.

The study used a sequential design to be able to stop the feasibility investigation in case of severe side effects. After inclusion of three consecutive patients a hold of recruitment was arranged until all three patients received at least 4 weeks of concomitant enoxaparin treatment. In absence of inacceptable toxicity by confirmation of the protocol committee the recruitment was continued until a minimum of 15 patients received at least 12 weeks concomitant enoxaparin treatment. Patients were followed up until death of any reason or lost to follow-up.

The trial was approved by the scientific and research ethics committee of our institution (Ethikkommission der Charité - Universitaetsmedizin Berlin). The investigation was conducted in accordance with the Declaration of Helsinki and Good Clinical Practice Guidelines and within the CONSORT guidelines. Furthermore, the national principles for the proper execution of the clinical examination of drugs (Bundesanzeiger No. 243 of 30.12.1987), the national regulations of the German drug law, and the German drug test guidelines were adhered. Trial registration: Clinical Trials NCT01945879*.*

### Eligibility criteria

Main inclusion criteria were: ambulatory patients with histologically confirmed APC, no previous radio- or chemotherapy, Karnofsky Performance Status (KPS) ≥ 60%; measurable tumour lesion confirmed by computed tomography (CT) or magnetic resonance imaging (MRI) within the last 14 days, no VTE within the last 2 years, adequate compliance and home residence within geographical proximity to our department (allowing an adequate follow-up), sufficient bone marrow function (leukocytes 3.5 × 10^9^/l, thrombocytes 100 × 10^9^/l), age ≥ 18 years.

Patients were excluded in case of pre-existing indication for anticoagulation, major bleeding events within the last 2 weeks or severe impairment of coagulation, active gastrointestinal ulcers or major surgery within the last 2 weeks, body weight < 45 kg or > 100 kg, pregnancy/lactation or insufficient contraception during study or severely impaired renal function (creatinine clearance < 30 ml/min).

All patients had to provide written informed consent.

#### Outcome measures

Primary investigation included the feasibility, the toxicity profile, and probable drug interactions. The first step was the analysis of safety data of the initial three patients after completing their first 4 weeks of combined treatment. On condition that there is no more than one patient with a toxicity of NCI-CTC grade 3/4 and no event of severe bleeding, the recruitment would be continued (protocol committee consensus). The final treatment feasibility and safety would be approved if the incidence of predefined severe grade 3/4 toxicities (NCI-CTC) would be no higher than 20% as well as the incidence of predefined severe, life threatening bleeding events would be no higher than 20% in a minimum of 15 patients under treatment duration of at least 12 weeks.

Secondary aims were overall survival (OS), influence of prognostic factors and the rate of symptomatic venous thromboembolic events and severe bleedings. Staging CT or MRI was performed at least every 12 weeks (earlier in case of suspected progression). According to safety aspects we documented severe bleedings in the event of short term decline of hemoglobin level (≥ 2 g/dl/48 h) in absence of other evidence (e.g. hemolysis) and/or the need for at least two red blood cell concentrates in case of confirmed blood loss and/or the clinical occurrence of serious apparent bleeding in parenchyma, muscle or cerebrum. The highest grade of a hematologically or non-hematologically toxicity during a cycle was recorded for the analysis.

#### Statistical methods

Statistical analyses were performed using R 3.01 with software package CMPRSK and SPSS software 19.0.

## Results

For the first three patients no intense toxicity, particularly no severe bleeding event, was observed within the required 4 weeks of treatment. Hence the recruitment was continued to get a minimum of 15 patients receiving combined chemotherapy and concomitant enoxaparin over a duration of at least 12 weeks.

As 15 patients completed the 12 weeks of treatment a total number of 19 patients were recruited (Table 1). 2 patients had progressive disease within the 12 weeks and decreased rapidly in performance status without qualifying for second line treatment. The remaining 17 patients were staged at 12 weeks, of which 15 patients had a stable disease and were getting further treatment with gemcitabine alone simultaneous with enoxaparin, whereas 2 patients had documented progressive disease and were switched to paclitaxel second-line regimen without enoxaparin treatment.

**Table 1:** Demographic and baseline characteristics

**Characteristic: N = 19 (100%)**	**Chemotherapy + Enoxaparin**
**Age – Years**	
Median [range]	59 [46 – 74]
**Sex – no. (%)**	
Female	6 (32)
Male	13 (68)
**KPS – no. (%)**	
60% - 70%	2 (11)
80% - 100%	17 (89)
**Stage – no. (%)**	
M0	6 (32)
M1	13 (68)
Liver and lung	1(5)
Liver	8 (41)
Lung	2 (11)
Other	2 (11)
**Primary cancer – no. (%)**	12 (63)
**Recurrent cancer – no. (%)**	7 (37)
Site of recurrence	
Liver	5 (71)
Local	2 (37)

**Figure 1:**
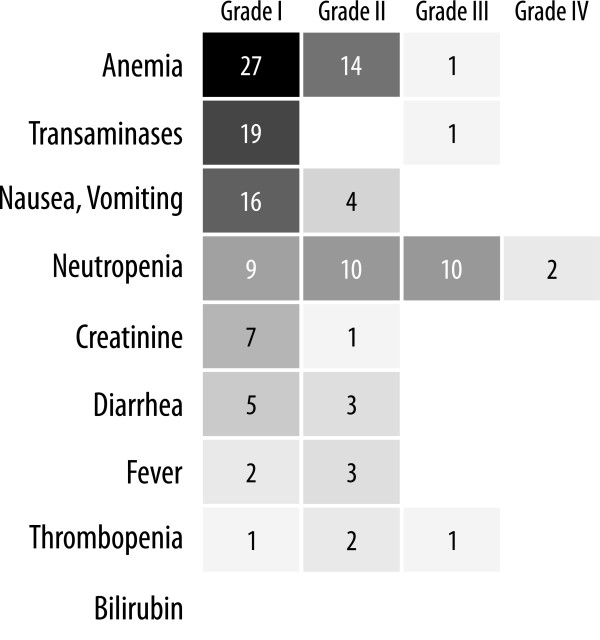
**Hematological and non-hematological toxicities (no. out of 179 cycles).** Numbers represent the count of treatment cycles with corresponding toxicity.

The analysis of cumulative toxicities was based on 71 (median 4/pt [range:2–4]) cycles of GFFC with enoxaparin and 108 (median 4/pt [range:0–18]) cycles of single-agent gemcitabine with enoxaparin (Figure 1). The incidence of NCI-CTC grade 3/4 toxicities was below 10%, most frequent side effect was neutropenia (grade 3: 12 cycles, grade 4: 2 cycles) and thrombocytopenia (grade 3: 1 cycle). During the course of GFFC the dose of cisplatin was reduced by 50% in 3 patients due to elevated serum creatinine levels, up to grade 2 in one patient. No grade 3/4 nausea or vomiting occurred. One case of symptomatic deep venous thrombosis with consecutive non-lethal pulmonary embolism was observed while receiving prophylactic LMWH treatment (5%). No patient stopped the subcutaneous heparin injections due to occurrence of local side irritations or generally inconvenience. No severe bleeding events as defined in the protocol were observed under treatment with enoxaparin as well as in a corresponding follow-up of 30 days.

**Figure 2:**
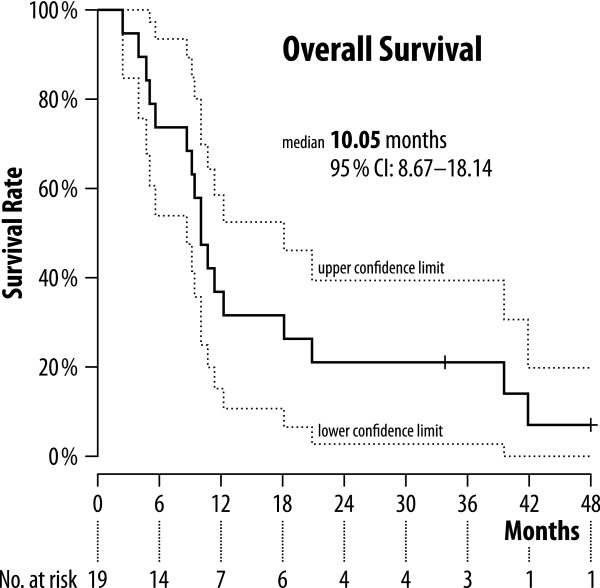
Kaplan-Meier plot for overall survival.

**Figure 3:**
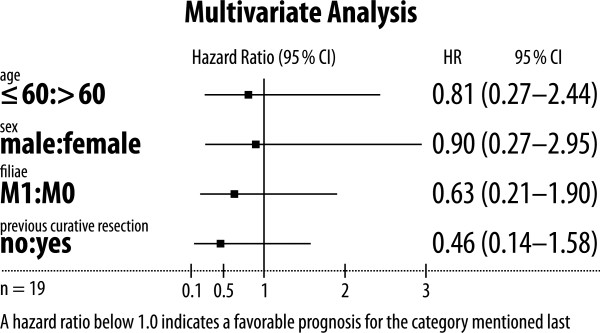
Forest plot of prognostic factors.

The median overall survival was 10.05 [95% CI: 8.67-18.14] months (see Figure 2). Two out of 19 patients were lost to follow-up and were censored at the time of last documented visit. The multivariate analysis of overall survival showed no significant effect on survival by a single characteristic (Figure 3). Best trend was observed for patients with previous curatively intended cancer resection followed by cancer recurrence versus patients with primary inoperable pancreatic adenocarcinoma.

## Discussion

This investigation was conducted to assess the use of intensified chemotherapy together with simultaneous application of heparin. The application of medical anticoagulation for cancer patients was still discussed over the last decade, even for inpatients or patients with confirmed thrombosis. The assumed higher incidence of severe bleeding events due to medical anticoagulation, especially in patients with advanced gastrointestinal malignancies, reduced the number of sufficient treated patients in the past and thus may lead to impaired overall survival [12,19].

This open label phase II feasibility investigation used intensified chemotherapy and simultaneous enoxaparin treatment in patients with first line chemotherapy. The dosage of enoxaparin to be used in our trial has been intensively discussed in terms of pre-existing prevention studies in other indications. Results from the PRINCE [20] and MEDENOX [21,22] studies demonstrated effective and well-tolerated VTE prevention in patients with severe cardiopulmonary disease using the LMWH enoxaparin. The given dosages in the MEDENOX trial were set at a high prophylactic level of 40 mg daily versus placebo and also in the PRINCE trial with 40 mg daily versus 5000 IU unfractioned heparin 3× daily. Thus 40 mg enoxaparin once a day was considered to be the minimal dose for primary symptomatic VTE prevention.

We used a safety step design to prevent patients from harmful increased toxicities due to the combination. The observed side effects were consistent with respect to the applied chemotherapy and were similar to those in our phase I trial [18]. We therefore continued the investigation without need of modification of the regimen. Even the addition of heparin does not lead to increased toxicities. We suspected a raise in the local bleeding rate in our advanced pancreatic cancer patients by potential cancer infiltration of the stomach or duodenum. Major bleeding rates for patients with solid cancer using warfarin were 42% and 14% for controls in 431 patients [23], in our safety trial we therefore decided to accept no higher rate than 20% using prophylactic anticoagulation with enoxaparin. Fortunately we did not observe any patient with severe bleeding under concomitant application of 40 mg enoxaparin daily. Only one patient had a thromboembolic event while getting enoxaparin (5%), whereas the supposed rate of thromboembolic events without heparin use would be 15-20% [11].

We further observed a slightly increased overall survival compared to gemcitabine monotherapy [2,9,24]. This finding and the open discussion about an independent effect of heparins with regard to metastasis and cancer growth has to be investigated by a randomized trial in a larger setting [17]. On the other hand this observed effect is commonly seen in phase II studies with the potential bias of unconscious patient selection and the addition of placebo effects [7,10]. But besides these considerations we can at least assume that the efficacy of the treatment is not negatively affected.

The finding of this investigation regarding feasibility and safety gives us confidence for using the researched combination in a larger trial to investigate the effect of heparin in a randomized setting.

## Conclusions

In summary, the addition of enoxaparin to GFFC is feasible, safe and does not appear to affect the activity or the toxicity profile of the chemotherapy regimen in patients with advanced pancreatic adenocarcinoma. To examine whether enoxaparin reduces the incidence of thromboembolic events and increases survival, we have initiated a randomized investigation study of GFFC (or single-agent gemcitabine for patients with impaired performance status) with or without enoxaparin (CONKO-004) [17].

## Competing interests

No potential conflicts of interest relevant to this report of our investigator initiated trial are present.

## Authors’ contributions

UP, AH, JMS, MS, MB and BG contributed to patient enrolment and to the collection and assembly of the data. HR and BD provided capacities for the trial. UP and HR drafted the manuscript. HR and UP were responsible for the study idea and design. HR, AH and UP were responsible for data analysis and interpretation. All authors provided final approval.

## Pre-publication history

The pre-publication history for this paper can be accessed here:

http://www.biomedcentral.com/1471-2407/14/204/prepub
